# IL-34 as a Novel Mediator Linking Vitamin D Deficiency with Osteoporosis and Knee Osteoarthritis

**DOI:** 10.3390/ijms262211090

**Published:** 2025-11-16

**Authors:** Nader Tarabeih, Ali Sleiman, Alexander Kalinkovich, Shai Ashkenazi, Adel Shalata, Gregory Livshits

**Affiliations:** 1 Department of Morphological Sciences, Adelson School of Medicine, Ariel University, Ariel 4070000, Israel; nadertar@gmail.com (N.T.); shaias@ariel.ac.il (S.A.); 2 Department of Nursing, The Max Stern Yezreel Valley College, Emek Yezreel 1930600, Israel; 3 Department of Orthopedics, Carmel Medical Center, Haifa 3436212, Israel; alisl@clalit.org.il; 4 Department of Anatomy and Anthropology, Gray Faculty of Medical and Health Sciences, Tel-Aviv University, Tel-Aviv 6905126, Israel; alexander.kalinkovich@gmail.com; 5 The Simon Winter Institute for Human Genetics, Bnai Zion Medical Center, The Ruth and Bruce Rappaport Faculty of Medicine, Technion, Haifa 3200003, Israel; adel.shalata@gmail.com

**Keywords:** body composition, fat mass, inflammation, cytokines, structural equation modeling

## Abstract

Vitamin D deficiency (VDD) is a widespread human condition closely associated with musculoskeletal disorders, involving alterations in body composition and systemic inflammation. In this cross-sectional study, 1075 adults were classified into a VDD (n = 304) group and a VD sufficient (VDS) group (n = 771). Body composition measurements, including the fat mass-to-weight ratio (FM/WT), were assessed using bioelectrical impedance analysis. Plasma levels of IL-9, IL-34, and MCP-1 were also measured. Data on osteoporosis (OP) and knee osteoarthritis (KOA) were collected. Regression analyses indicated that FM/WT was independently associated with VDD, which in turn was linked to elevated IL-34 levels. Individuals with VDD had a significantly higher prevalence of OP and KOA compared with those with VDS. Structural equation modeling confirmed and quantified these associations, suggesting that FM/WT is a significant predictor of VDD status (β = 0.305, 95% CI: 0.231–0.367). VDD is directly associated with elevated IL-34 levels (β = 0.353, 95% CI: 0.308–0.398), while IL-34 levels, in turn, are a possible cause of KOA (β = 0.573, 95% CI: 0.460–0.687) and OP (β = 0.433, 95% CI: 0.329–0.536). Our data clearly demonstrate existence of a physiological-pathological continuum: obesity-VDD-IL-34 and both OP and KOA.

## 1. Introduction

Vitamin D (VD) is a steroid hormone that plays a central role in calcium and phosphate homeostasis, skeletal development, and immune regulation [1,2]. Its synthesis begins in the skin upon exposure to ultraviolet B radiation, followed by sequential hydroxylations in the liver to 25-hydroxyvitamin D [25(OH)D], the main circulating form, and in the kidney to 1,25-dihydroxyvitamin D [1,25(OH)_2_D], the biologically active metabolite [1,2]. The active metabolite binds to the nuclear VD receptor (VDR), which is widely expressed in bone, cartilage, and immune cells, linking VD status to musculoskeletal and immune function [3].

Changes in body composition are associated with circulating VD levels, because the adipose tissue serves as a major storage site for VD [4,5]. Consequently, higher fat mass (FM) can sequester VD, reducing its bioavailability in the circulation. Studies consistently reported an inverse association between circulating 25(OH)D levels and obesity, particularly with higher body mass index (BMI) and central adiposity [6,7]. Reduced muscle mass was also associated with impaired VD metabolism and bioavailability [8,9]. Moreover, recent studies indicate that VD deficiency (VDD) is associated with adverse metabolic profiles, including dyslipidemia characterized by elevated total plasma cholesterol (TC) and triglycerides (TG) [10,11]. However, the results remain inconsistent, and the associations between VD status, body composition, and lipid levels are still under debate [12].

VDD is highly prevalent worldwide and is associated with a wide spectrum of chronic diseases [13,14]. While it is classically associated with rickets and osteomalacia [15], accumulating evidence highlights its broader significance in inflammation-mediated disorders, including autoimmune diseases, cardiovascular and metabolic conditions, and bone disorders [16,17,18,19]. Among these, the association between VDD and osteoporosis (OP) is one of the most extensively studied [20]. Increasingly, OP is considered a chronic inflammatory condition wherein dysregulated immune signaling drives bone loss [21]. Pro-inflammatory cytokines, such as IL-1β, IL-6, IL-17, TNF-α, and RANKL, promote osteoclast differentiation and activation, thereby enhancing bone resorption. Conversely, anti-inflammatory cytokines, including IL-10, IL-4, and IL-13, contribute to osteoprotection by suppressing osteoclastogenesis and supporting T regulatory and Th2 responses [22,23]. VDD disrupts the cytokine balance, exacerbating bone degradation through both endocrine and immune-mediated mechanisms [24,25]. In addition, VDD impairs calcium absorption and triggers secondary hyperparathyroidism, which accelerates bone turnover and resorption [26,27,28].

Emerging evidence suggests a role for VDD also in the pathogenesis of osteoarthritis (OA), the most prevalent musculoskeletal disorder worldwide, characterized by cartilage degradation, subchondral bone restructuring, synovial inflammation, and chronic pain [29]. VDD has been associated with increased prevalence, pain severity, and accelerated radiographic progression of knee OA (KOA) in multiple epidemiological studies [30]. VD’s roles in OA pathogenesis are supported by its effects on cartilage, bone, and synovial inflammation [29]. Several studies have reported an association between low circulating VD levels and increased prevalence and severity of radiographic spinal OA, including disk space narrowing and osteophyte formation [31,32,33,34,35]. Although the underlying mechanisms remain incompletely understood, chronic low-grade inflammation and impaired subchondral bone remodeling, both influenced by VD status, are possible contributors to OA etiology [36,37,38,39].

Several inflammatory markers and derived indices have been implicated in musculoskeletal pathology in the context of VDD. Classical markers, such as high-sensitivity C-reactive protein (hs-CRP), which reflects overall inflammatory burden [40], and composite indices like the Systemic Immune-Inflammation Index (SII), both showed significant inverse correlations with serum 25(OH)D levels [41].

Recent evidence highlights the role of IL-9–secreting immune cells in a broad range of inflammatory and autoimmune diseases [42,43]. Notably, postmenopausal OP patients exhibit increased frequencies of IL-9-secreting Th9 cells, and in ovariectomized mice, IL-9 has been shown to promote osteoclastogenesis [44]. Moreover, serum IL-9 concentrations in OA patients correlate with both disease severity and hs-CRP levels [45].

Similarly, the pro-inflammatory chemokine MCP-1/CCL2 enhances osteoclastogenesis [46], and its elevated serum levels have been reported in OP patients, where they associate with disease severity [47]. A meta-analysis confirmed increased circulating levels of MCP-1/CCL2 in OA patients [48]. Furthermore, in vitro studies demonstrated its capacity to induce chondrocyte degradation [49]. Another cytokine of interest in this context is IL-34, which not only promotes osteoclastogenesis and inflammatory signaling but also exerts immunoregulatory effects [50]. Elevated plasma IL-34 levels have been reported as biomarkers in periodontal disease, KOA, psoriatic arthritis, and rheumatoid arthritis [51,52,53]. In our recent study, we observed significantly higher, age-independent plasma IL-34 levels in women with OP compared with non-osteoporotic controls [54]. Nevertheless, although VDD on the one hand, and IL-9, MCP-1/CCL2, and IL-34 on the other hand, have been implicated in the pathogenesis of OP and OA, and despite clinical and experimental evidence showing a correlation between VDD and elevated circulating cytokine levels, the potential link between VDD and these inflammatory factors in the etiology of musculoskeletal diseases remains largely unexplored.

Given this background, the present study was designed with two primary aims. First, we sought to evaluate whether body composition measures, especially FM, are risk factors associated with VDD. Second, we investigated the associations among VDD, inflammatory markers, and musculoskeletal outcomes, focusing on OP and KOA, with an emphasis on their potential interactions between VDD and cytokines. We hypothesized that VDD would be linked to a pro-inflammatory immune milieu, reflected by elevated levels of inflammatory markers and cytokines, which may contribute to the pathogenesis of both OP and KOA.

## 2. Results

### 2.1. Descriptive Characteristics of the Study Sample

The study sample consisted of 1075 participants (487 males and 588 females). Among them, 304 individuals (28.3%) were VDD, whereas 771 (71.7%) had vitamin D sufficient (VDS) levels. The prevalence of VDD was significantly higher in females compared to males (38.4% vs. 16.0%; *p* < 0.00001). The mean serum 25(OH)D concentration in the total sample was 31.3 ± 19.5 nmol/L (range: 6.2–118 nmol/L). Participants with VDD displayed significantly lower levels (20.0 ± 10.4 nmol/L) compared to those with VDS (43.2 ± 19.9 nmol/L; *p* < 0.00001). When stratified by sex, females had significantly lower VD levels than males (24.9 ± 14.8 nmol/L vs. 43.2 ± 21.6 nmol/L; *p* < 0.001). In subsequent analyses, comparisons of all other variables were conducted while adjusting for age and sex.

### 2.2. Group Comparisons by VD Status

Basic comparative descriptive statistics and group comparisons are presented in Table 1. Individuals with VDD were significantly older than VDS (45.7 ± 0.8 vs. 42.0 ± 0.5 years, *p* = 0.00006), but displayed no significant differences in BMI and waist–hip ratio (WHR) distributions. In contrast, the fat mass/weight ratio (FM/WT) was significantly higher in the VDD group (0.362 ± 0.004 vs. 0.299 ± 0.002, *p* = 0.001).

Comparison of the lipid profile revealed a significant difference between the groups (*p* = 0.01), with only the ratio of total cholesterol to high-density lipoprotein cholesterol (TC/HDL-C) remaining significant after adjustment for age and sex. However, it became insignificant after Bonferroni correction.

**Figure 1:**
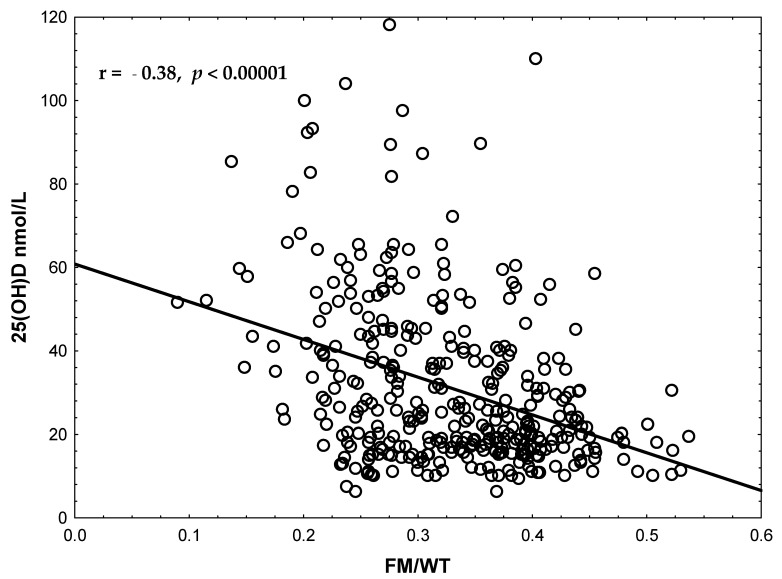
Pearson correlations between serum levels of 25(OH)D and FM/WT. The correlation coefficients were computed after adjustment for age and sex.

Among inflammatory markers, IL-34 levels were markedly elevated in VDD individuals, reaching nearly an eight-fold difference compared with VDS individuals (567.7 ± 68.5 vs. 72.6 ± 5.4 pg/mL; *p* < 0.000001). hs-CRP levels were also higher in the deficient group (1.95 ± 0.31 vs. 0.93 ± 0.13 mg/L; *p* = 0.004). In contrast, IL-9, MCP-1, and SII showed no significant differences between the groups and were not further examined. Musculoskeletal outcomes were statistically highly significantly associated with VD status. The prevalence of OP was 9.8% among participants with VDD compared to 1.7% in the VDS group (*p* < 0.00001), and KOA was observed in 15.7% of VDD individuals compared to 4.3% in the VDS group (*p* < 0.00001). These associations remained significant after multiple testing correction. In accordance with this was the clear pattern of the inverse correlation between FM/WT and serum 25(OH)D concentrations (r = −0.38, *p* < 0.001, Figure 1), which persisted after adjustment for age and sex.

### 2.3. Regression Analyses of VD Status and IL-34 Levels

Next, we conducted multiple logistic regression analyses of VDD status to evaluate the extent of the independent association with the body composition measure (FM/WT) that remained significant in the univariate comparisons, simultaneously adjusting for age and sex differences between the individuals (Table 2). The results of this analysis suggested that FM/WT was significantly and independently associated with VDD (OR = 1.37, 95% CI: 1.09–1.72; *p* = 0.006). Both sex and age were also significantly associated (probable predictors of) with VDD: females had more than a four-fold higher risk compared with males (OR = 4.11, 95% CI: 2.56–6.59; *p* = 4.4 × 10^−8^), and increasing age was associated with higher odds for VDD (OR = 1.38, 95% CI: 1.14–1.66; *p* = 0.0005).

Next, we assessed the association of VD status with the IL-34 levels. Multiple linear regression was conducted with IL-34 plasma levels as the dependent variable and VD status, age and sex as independent predictors (Table 3). The results of this analysis suggested that VDD emerged as statistically the strongest independent predictor of IL-34 levels (β = 0.53, SE = 0.05, *p* = 6.99 × 10^−23^), whereas age and sex were not significantly associated. These findings confirm that elevated IL-34 is specifically linked with VDD, independent of demographic factors.

### 2.4. Associations Between VD Status, OP, and KOA

We next examined the associations between VD status and musculoskeletal outcomes by implementing chi-square (Table 4). VDD was strongly associated with both OP (χ^2^ = 38.11, Phi = 0.19, *p* < 0.00001) and KOA (χ^2^ = 43.15, Phi = 0.20, *p* < 0.00001). In addition, OP and KOA were significantly correlated with each other (χ^2^ = 21.07, Phi = 0.14, *p* < 0.00001). Notably, all associations remained significant after adjustment for age and sex.

These findings indicate that VDD is not only linked to altered body composition, specifically FM/WT, and IL-34 marker (Table 1), but also to clinically relevant skeletal outcomes, reinforcing its potential role in the pathophysiology of OP and KOA.

### 2.5. Structural Equation Modeling (SEM)

**Figure 2:**
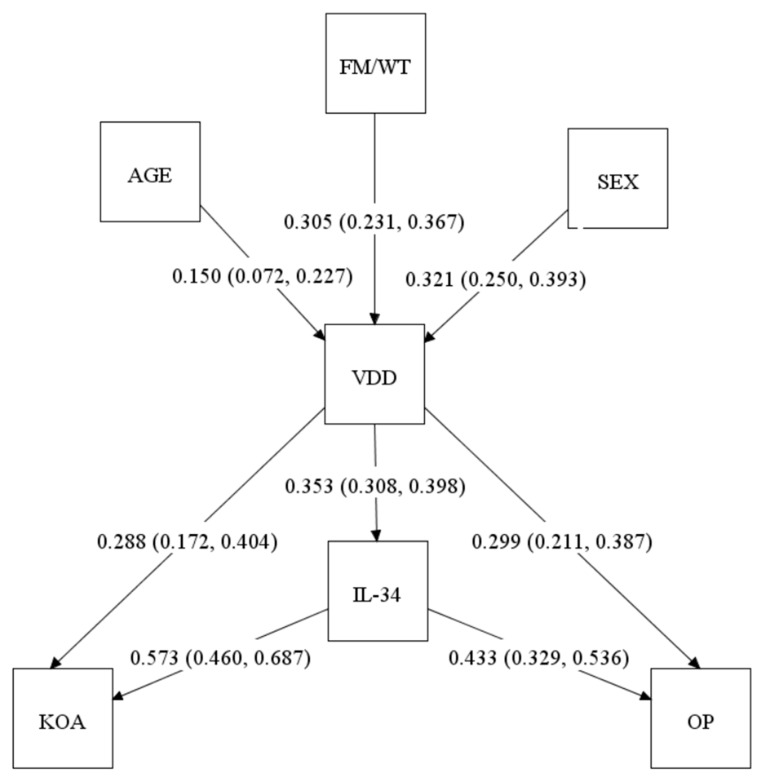
Path diagram, generated by structural equation model, illustrating associations between age, sex, fat mass-to-weight ratio (FM/WT), vitamin D deficiency (VDD), IL-34, osteoporosis (OP), and knee osteoarthritis (KOA). The associations between the variables are presented as standardized path coefficients (β) with corresponding 95% confidence intervals (shown in parentheses), i.e., β (lower CI, upper CI). The β coefficient represents the standardized strength and direction of the association between two variables, whereas the two numbers in the CI denote the lower and upper bounds of the 95% CI. All reported paths are statistically significant (*p* < 0.05). Age, sex, and FM/WT were modeled as predictors of VDD. VDD was directly associated with increased IL-34 levels, OP, and KOA, and indirectly associated with OP and KOA via IL-34, indicating partial mediation of these relationships.

To integrate all the observed associations into a single framework, we applied SEM methods incorporating age, sex, FM/WT, VD status, IL-34, OP, and KOA (Figure 2). The model showed that FM/WT (β = 0.305, 95% CI: 0.231–0.367), age (β = 0.150, 95% CI: 0.072–0.227), and sex (β = 0.321, 95% CI: 0.250–0.393) were significant predictors of VDD, consistent with the logistic regression findings (Table 2). In turn, VDD was directly associated (likely affecting) with IL-34 levels (β = 0.353, 95% CI: 0.308–0.398), OP (β = 0.299, 95% CI: 0.211–0.387), and KOA (β = 0.288, 95% CI: 0.172–0.404).

However, IL-34 levels, in turn, were significantly associated (likely affecting) with KOA (β = 0.573, 95% CI: 0.460–0.687) and OP (β = 0.433, 95% CI: 0.329–0.536), supporting its role as a mediator. The results of SEM clearly suggested that IL-34 elevated the risk for OP and KOA.

Combining these results, with help of path analysis (Mplus software, version 8.11), we created a path diagram suggesting specific direct and indirect relationships between the studied variables (Figure 2). The diagram suggests direct effects (associations) of age, sex, and FM/WT on the occurrence of VDD, which in turn influences both KOA and OA diagnoses directly and indirectly through IL-34. In this analysis, IL-34 emerges as an important mediator of VDD effect. However, the presence of the direct VDD effects may also indicate the involvement of other, as yet unidentified, factors mediating the association between VDD and musculoskeletal conditions.

### 2.6. X-Ray Images of KOA

**Figure 3:**
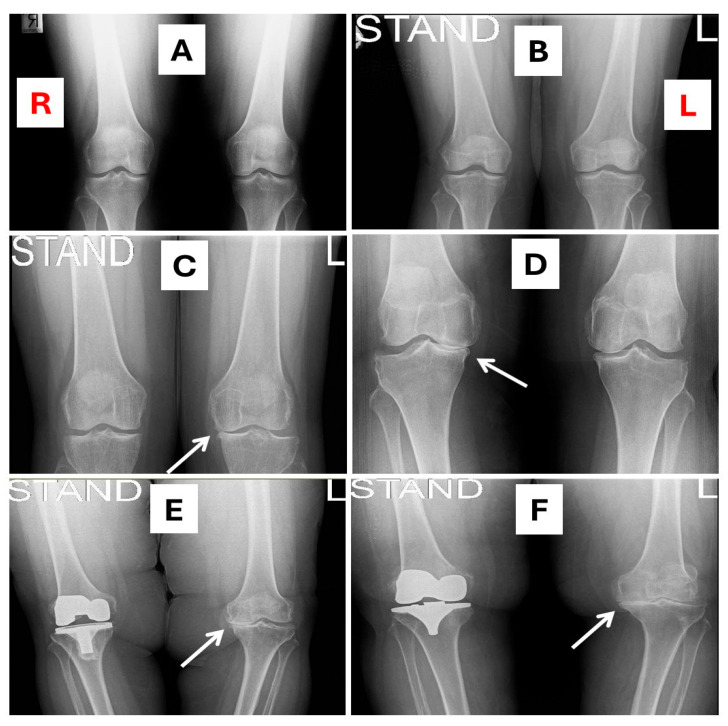
Illustrative clinical cases with biomarker profiles and knee radiographs. The side of each knee, right (R) or left (L), is clearly labeled, and arrows indicate the side or area of special clinical relevance (such as severe deformity). (**A**) Healthy control: 55-year-old woman with FM/WT (0.31), VDD (25(OH)D = 37 nmol/L), and low IL-34 plasma levels (72.9 pg/mL). Radiographs show preserved joint spaces, smooth margins, and no osteophytes, sclerosis, or deformity. (**B**) Early OA: 50-year-old woman with FM/WT (0.32) VDD (25(OH)D = 26 nmol/L) and elevated IL-34 plasma levels (150 pg/mL). Radiographs reveal early osteophytes at tibial spines and femoral margins with preserved joint spaces and no definite narrowing. (**C**) Mild OA: 51-year-old woman with FM/WT (0.33), VDD (25(OH)D = 21 nmol/L), and elevated IL-34 plasma levels (350 pg/mL). Radiographs demonstrate definite osteophytes with mild medial joint-space narrowing and early subchondral sclerosis. (**D**) Severe OA: 57-year-old woman with FM/WT (0.45), VDD (25-OH = 15 nmol/L), and markedly elevated IL-34 plasma levels (3400 pg/mL). Radiographs show severe narrowing of the joint space, large osteophytes, and marked sclerosis. (**E**) Post–unilateral knee replacement: 60-year-old woman with FM/WT (0.45), VDD (25(OH)D = 16 nmol/L), and elevated IL-34 plasma levels (2850 pg/mL). Radiographs show right total knee arthroplasty with prosthesis, while the left knee demonstrates advanced osteoarthritic changes with marked narrowing, sclerosis and varus deformity. (**F**) Post–unilateral knee replacement: 62-year-old woman with FM/WT (0.52), VDD (25(OH)D = 12 nmol/L), and very high IL-34 plasma levels (4100 pg/mL). Radiographs show right total knee arthroplasty with prosthesis, whereas the left knee exhibits severe osteoarthritic changes with joint space narrowing, sclerosis and varus deformity. Abbreviations: FM/WT, fat mass-to-weight ratio; VDD, vitamin D deficiency.

Figure 3 shows representative radiographic examples of KOA manifestations, contrasting affected individuals with a subject exhibiting healthy knee joints. This visual comparison demonstrates the combined association of an elevated FM/WT ratio, VDD, increased IL-34 plasma levels, and advanced KOA stages.

## 3. Discussion

### 3.1. Main Results of the Study in Relation to Underlying Molecular Mechanisms

This study provides new insights into the complex relationship between VDD, obesity, IL-34, OP and KOA. Our main findings are (1) obesity, reflected by elevated fat mass, is strongly associated with VDD, and likely predispose to it, along with age and female sex; (2) VDD was significantly associated with IL-34 levels, suggesting a potential direct link between VD status and IL-34 expression; (3) IL-34 elevation is associated with both OP and KOA and most likely contributes to their etiology.

This combined association between fat mass, VDD, high IL-34 plasma levels, and advanced stages of KOA is clearly illustrated in Figure 3. This figure demonstrates that a progressive worsening of radiographic severity of KOA is associated with elevated concentrations of IL-34 and with declining VD levels, paralleled by body fat mass increase, thereby reinforcing the translational link between obesity, biomarker dynamics and clinical pathology.

Finally, our path analysis also suggests an additional path from VDD to both KOA and OP, likely mediated by factors not examined in this study. Together, these findings emphasize that VDD operates as a multifaceted risk factor in musculoskeletal disorders.

The first result of this study underscores the well-documented link between obesity and VDD [13,14,55]. Mechanistically, VD, a lipophilic hormone, becomes sequestered in adipose tissue, lowering its circulating bioavailability [5,56]. Obesity is also accompanied by chronic low-grade inflammation, driven by adipocyte-derived cytokines and macrophage infiltration [57,58], which, in turn, impairs VD metabolism [59].

To the best of our knowledge, our second finding—the significant association between VDD and elevated IL-34 plasma levels—is the first to be reported. VD is very likely to affect IL-34 via several molecular pathways. Thus, via its receptor, VDR, VD inhibits NF-κB signaling [24,60]. On the other hand, NF-κB can induce IL-34 expression in monocytes, macrophages, and fibroblast-like synoviocytes [61,62]. VD also attenuates activation of STAT3 (signal transducer and activator of transcription 3), a protein that plays a crucial role in regulating cell growth, differentiation, immune responses, and apoptosis [63,64], and is a transcriptional driver of IL-34 in immune cells [65]. Consequently, it is plausible to suggest that VDD removes two inhibitory mechanisms—the repression of NF-κB and STAT3—thereby promoting IL-34 transcription.

The third key result in this study demonstrates that IL-34 is significantly associated with both OP and KOA. The possible mechanism underlying this association is of interest and significance. Previous studies on this subject are limited; however, the few that exist are consistent with our findings. In the context of OP, it has been shown that IL-34 is expressed by osteoclasts and stimulates RANKL-induced osteoclastogenesis by promoting the proliferation, adhesion, differentiation, and fusion of osteoclast progenitors, while also enhancing their resorptive activity [66,67], primarily through NF-κB signaling pathways [68]. Consistent with these in vitro findings, in vivo administration of IL-34 in mice reduces trabecular bone mass [68]. Conversely, IL-34 knockdown [69] or neutralization with specific antibodies [70] suppresses osteoclastogenesis. We have recently reported, in a preliminary study, that plasma IL-34 levels are significantly and independently associated with OP, suggesting that circulating IL-34 levels could serve as a systemic biomarker for OP and BMD status [54].

A possible association of IL-34 and OA has also been reported. For example, elevated IL-34 levels have been observed in plasma and synovial fluid in patients with KOA, particularly in advanced disease stages, and were associated with both radiographic and symptomatic severity [51,71], similar to our present findings (e.g., Figure 3).

IL-34 is produced by a variety of cells, including monocytes, macrophages, microglia, endothelial cells, fibroblasts, neurons, hepatocytes, and epithelial cells [72,73]. It is also expressed by keratinocytes in the skin and synovial fibroblasts, where its expression can be induced by pro-inflammatory cytokines such as TNFα and IL-1β. Recent evidence further indicates that adipose tissue constitutes an important source of IL-34. Elevated circulating IL-34 concentrations have been reported in obese individuals compared with lean controls, and IL-34 expression increases during adipogenesis, where it promotes lipid accumulation and adipocyte differentiation [74]. These findings suggest that adipose tissue-derived IL-34 may serve as a mechanistic link between metabolic inflammation and joint or bone degeneration, consistent with the associations observed in our study.

Our findings propose that VDD stimulates the elevation of IL-34 circulating levels. Mechanistically, VD is known to suppress pro-inflammatory cytokine production [75] and regulate macrophage function [76]. Its deficiency could therefore shift the immune balance toward a pro-inflammatory phenotype [77], favoring IL-34 production. In turn, elevated IL-34 may promote osteoclast differentiation and cartilage breakdown, providing a plausible mechanistic link between VDD, OP, and OA through an inflammatory axis.

Although our findings and proposed model suggest that VDD may upregulate IL-34, the effect of VD supplementation on IL-34 expression remains largely unexplored. To date, no human studies have directly evaluated whether VD replacement reduces circulating IL-34 levels. Interestingly, in neural models, VD was shown to increase IL-34 expression via VDR-dependent mechanisms, potentially exerting neuroprotective and anti-inflammatory effects in the central nervous system [78]. These results imply that the regulatory relationship between VD and IL-34 may be tissue-specific—stimulatory in neural cells but possibly suppressive in peripheral inflammatory or adipose tissues.

### 3.2. VD Supplementation and Musculoskeletal Diseases

It is well established that VDD is strongly associated with bone loss, suggesting that supplementation could mitigate this effect. Indeed, numerous studies on this aspect reported significant benefit in terms of bone healing [79,80,81]. The corresponding results of the present study are consistent with the previous reports concerning VDD—bone loss association [29,30,82,83]. Our study also clearly shows a significant association between VD levels and OA diagnosis. However, evidence regarding whether supplementation of VD reduces cartilage degradation or alleviates pain in OA remains conflicting. Reported outcomes range from rare protective effects [84] to predominantly ineffective results [85,86,87,88]. Nevertheless, several studies reported a significant association between low VD levels and KOA [89]. One study, particularly relevant to the present context, found that patients with KOA had lower levels of VD and higher levels of inflammatory biomarkers, specifically IL-1β, TNFα, hs-CRP, and NF-κB p65, compared to healthy controls [90]. A recent meta-analysis, based on 25 studies investigating the association between VDD and KOA, showed that more than 50% of the patients had VDD, with especially high prevalence in Europe and among individuals with obesity [91]. The authors concluded that targeted screening for VDD in KOA patients is recommended, which was one of the aims of the present study.

The fourth finding, the positive correlation between IL-34 levels and X-ray-defined OA severity, further supports IL-34’s clinical relevance. The observed correlation suggests that systemic IL-34 levels reflect not only the presence but also the progression and structural severity of OA. Together, our findings delineate a pathophysiological pathway: Obesity → VDD → IL-34 upregulation → bone and joint degeneration. The scientific novelty of this study lies in identifying IL-34 as a mechanistic link in this axis, offering an explanation concerning the VDD association with musculoskeletal pathology. These findings highlight the potential clinical utility of VDD and IL-34 as accessible biomarkers for the early identification of individuals at high risk for OP and OA, thereby facilitating timely preventive or therapeutic interventions.

Despite these promising insights, some limitations must be acknowledged. The cross-sectional design of this study precludes any conclusions about causality. It remains uncertain whether VDD drives IL-34 elevation, whether IL-34 modulates VD metabolism, or whether both are secondary to a shared underlying factor such as systemic inflammation or lifestyle-related factors. The reported relationship between VDD and IL-34 in the context of obesity and musculoskeletal disorders requires longitudinal and mechanistic studies. Furthermore, our analysis, while controlled for key confounders, cannot rule out influences of genetic variation in VD metabolism [88,92,93,94], dietary intake [95], or environmental exposures [15], including seasonal variation and sunlight exposure, which were not directly quantified. Another limitation of this study is that both KOA and OP were used as binary variables, based on the orthopedist’s assessment as “diagnosed” vs. “not diagnosed”. Therefore, it would be of interest to further examine the extent of the corresponding associations, considering specific radiographic signs, such as joint space narrowing. Future longitudinal studies incorporating standardized imaging-based grading and detailed clinical phenotyping are warranted to validate and extend these findings.

In conclusion, the findings of the present study, together with existing evidence, highlight important implications for clinical practice. The integration of VD status with IL-34 measurement may improve early identification of individuals at elevated risk for OP and KOA. VDD screening is already feasible in routine clinical and community settings, and the addition of IL-34 as a circulating biomarker could help distinguish patients in whom inflammatory mechanisms drive musculoskeletal deterioration. This dual approach supports more personalized prevention strategies: ensuring adequate VD and calcium intake broadly, while considering targeted anti-inflammatory interventions in those with elevated IL-34. Such strategies could ultimately contribute to reducing the burden of OP fractures and OA-related disability in at-risk populations, with a major impact on health.

## 4. Materials and Methods

### 4.1. Study Sample and Ethics

This cross-sectional study included 1075 participants (men and women, aged 18–70 years) recruited consecutively between January 2016 and January 2024 from outpatient clinics in Sakhnin, Israel. These clinics provide general healthcare services, including primary and family medicine. The study population is ethnically and culturally homogeneous, consisting of Israeli Arabs. Participants were recruited as part of a larger cohort originally established to investigate musculoskeletal and metabolic health. Individuals attending the clinics during the recruitment period were invited to participate regardless of health status. Participants were excluded if they met any of the following conditions: age < 18 years, pregnancy, known autoimmune or chronic inflammatory diseases (e.g., rheumatoid arthritis, lupus, inflammatory bowel disease), malignancy, severe renal or hepatic disease, or endocrine disorders affecting bone metabolism (e.g., hyperthyroidism, hyperparathyroidism, Cushing’s syndrome). All participants provided written informed consent and underwent standardized clinical screening by trained nurses, including anthropometric evaluation and blood sampling. The study protocol was approved by the IRB-Helsinki Committee of Meir Medical Center (Approval No. 042/2013K) and the Ethics Committee of Tel Aviv University (Approval No. 0000265).

### 4.2. Demographic, Anthropometric, and Body Composition Assessment

Data collection details of the same cohort have been described in our earlier publications [96,97,98]. Briefly, anthropometric measurements consisted of height (cm), weight (kg), BMI in kg/m^2^, and WHR. Body composition was assessed by the bioelectrical impedance analysis (BIA) method using the BIA101 device (Akern Bioresearch, Florence, Italy) [99]. BIA estimates body compositions based on the differing electrically conductive properties of various tissues. It measures the impedance, or opposition, to a flow-level alternating electrical current through body fluids, which are primarily contained in lean tissues. The analyzer operates at a constant frequency current (50 kHz, 90 µA). Each pair of adhesive skin electrodes is placed on the hand and foot on one side, typically the right side of the body, and connected to the instrument by an electrode cable set, with the subject in supine position [100]. BIA is a safe, reliable, simple, accurate, and cost-effective technique for assessing multiple body composition parameters. Importantly, its accuracy is comparable to DXA, while avoiding radiation exposure [99]. In this study, the BIA101 device was used to evaluate fat mass (FM) and appendicular skeletal muscle mass (ASMM) expressed in kilograms, as derived from the impedance vector. Further details on BIA technology are available in numerous publications over the past two decades (e.g., [101]). Since both FM and ASMM are significantly correlated with total body mass [102], they were standardized relatively to body weight, yielding the FM/WT and ASMM/WT ratios, used in the present statistical analyses.

### 4.3. Measurement of VD and Cytokine Circulating Levels

Serum VD levels were measured by a standard automated chemiluminescence immunoassay, as employed in routine clinical laboratories within Clalit Health Services [103]. The cutoff used to divide VD levels was a circulating 25(OH)D concentration of 25 nmol/L. Individuals with serum 25(OH)D < 25 nmol/L were classified as deficient and assigned to the VDD group, whereas those with concentrations ≥ 25 nmol/L were classified as sufficient and assigned to the VDS group [104].

Serum levels of hs-CRP were also measured by implementing standard methodology [105,106].

In addition to these routine clinical measurements, plasma levels of selected cytokines were measured to assess inflammatory pathways relevant to the study. Venous blood samples were collected from all study individuals after an overnight fast. Within one hour of collection, they were centrifuged for 15 min at 1800× *g* at 4 °C. Plasma fractions were separated and stored in aliquots at −70 °C. Levels of biomarkers were determined by ELISA using the DuoSet kits (R&D Systems, Minneapolis, MN, USA) according to the manufacturer’s protocols. The detection limits were as follows: 39.1 pg/mL for IL-9; 62.5 pg/mL for IL-34, 15.6 pg/mL for MCP-1/CCL2. The intra- and inter-assay coefficients of variation were between 5.7 and 8.6%. Before statistical analysis, the original measurements with distributions that significantly deviated from normality were log-transformed to approximate a normal distribution.

### 4.4. Blood Glucose Levels, Lipid Profile, and Blood Count

Fasting venous blood samples were obtained for complete blood count, glucose, and lipid profile measurements, including TC, TG, high-density lipoprotein cholesterol (HDL-C), and low-density lipoprotein cholesterol (LDL-C). The complete blood count included lymphocyte, monocyte, neutrophil, and platelet counts, which were used to calculate the SII, defined as: SII = (platelets × neutrophils)/lymphocytes ratio [41].

### 4.5. OP and KOA Assessment

OP was diagnosed using dual-energy X-ray absorptiometry (DXA); following World Health Organization (WHO) criteria, a T-score of ≤−2.5 at either the lumbar spine or femoral neck was considered diagnostic [107]. KOA was diagnosed according to the clinical and radiographic criteria of the American College of Rheumatology, and each case was confirmed by physician evaluation to ensure diagnostic accuracy [108,109].

### 4.6. Study Design and Statistical Analyses

The study design included two major stages: first, a comparative examination of the two groups under the study (VDS vs. VDD); and second, multivariable analyses including regression models and structural equation modeling (SEM) to assess predictors of VD status and musculoskeletal outcomes.

Prior to statistical analysis of the data, we computed basic descriptive statistics of all the study variables to identify possible outliers and to examine the variables’ distributions. Group differences (VDS vs. VDD) were compared using analysis of covariance (ANCOVA), controlling for age and sex. Categorical variables were presented as counts and percentages, with between-group differences assessed using the chi-square test. To adjust for the multiple testing bias, the Bonferroni correction was applied [110]. Further steps of analysis included the computation of paired Pearson correlations between serum levels of VD and all the quantitative variables of interest.

Next, regression analyses were conducted to evaluate the extent of associations of several covariates with VD status and inflammatory cytokines. The variables (e.g., FM/WT) that remained significant in the univariate comparisons, after Bonferroni correction, were entered into a multiple logistic regression model with VD status (VDS vs. VDD) as the dependent variable. Using the stepwise forward selection option, and for each covariate that remained significant in the final model, odds ratios (ORs) with 95% confidence intervals (CIs), β coefficients, and standard errors (SEs) were reported. Subsequently, IL-34 and hs-CRP (which were the only inflammatory markers that remained significant after adjustments and Bonferroni correction) were further analyzed as dependent variables using multiple linear regression. In both models, VD status (VDS vs. VDD) was included as a covariate along with age and sex. Standardized β coefficients with standard errors (SEs) were reported to assess the strength and direction of associations. In both models, all the variables in the analysis were standardized prior to statistical analysis.

We then examined the association between the VD status and both musculoskeletal outcomes, specifically, OP and KOA. The statistical significance of these pairwise relationships was examined by chi-square tests and the corresponding Phi coefficients, with adjustment for age and sex. All the above statistical analyses were performed using Statistica 64 (TIBCO Software Inc., Palo Alto, CA, USA; Version 14.0.1).

Finally, a path model implementing SEM was specified in Mplus (version 8.11) [111] to evaluate direct and indirect associations among all the variables that showed significant associations with VD status in the previous analyses. Path analyses were performed using robust maximum likelihood ratio (MLR) estimation, with missing data handled via full information maximum likelihood (FIML). Indirect effects were evaluated using bias-corrected bootstrapped 95% confidence intervals. Standardized path coefficients (β) with 95% CIs were estimated. Continuous predictors were standardized before analysis.

## Figures and Tables

**Table 1:** Basic descriptive statistics and comparison of the assessed variables between vitamin D sufficient (VDS) and vitamin D deficient (VDD) groups *.

Study Group/ Variable	VDS (n = 771) (VD Levels ≥ 25 nmol/L)	VDD (n = 304) (VD Levels < 25 nmol/L)	*p*-Value
Age (y)	42.008 ± 0.498	45.718 ± 0.762	0.00006 ^#^
BMI (kg/m^2^)	27.851 ± 0.182	29.214 ± 0.349	NS
WHR	0.905 ± 0.003	0.904 ± 0.005	NS
FM/WT	0.299 ± 0.002	0.362 ± 0.004	0.001 ^#^
ASMM/WT	0.271 ± 0.001	0.246 ± 0.002	NS
Glucose (mg/dL)	96.657 ± 1.054	98.154 ± 1.423	NS
TG (mg/dL)	126.100 ± 3.267	125.926 ± 4.455	NS
TC (mg/dL)	177.263 ± 1.353	180.816 ± 2.180	NS
TC/HDL-C	4.109 ± 0.049	3.996 ± 0.071	0.01
IL-9 (pg/mL)	205.367 ± 61.127	204.221 ± 89.217	NS
IL-34 (pg/mL)	72.585 ± 5.439	567.698 ± 68.486	0.0000001 ^#^
MCP-1 (pg/mL)	3.823 ± 0.023	3.893 ± 0.039	NS
hs-CRP (mg/L)	0.925 ± 0.127	1.948 ± 0.305	0.004
SII	513.944 ± 16.170	516.601 ± 19.966	NS
OP, %	1.7% (13)	9.8% (30)	<0.00001 ^#^
KOA, %	4.3% (33)	15.7% (48)	<0.00001 ^#^

* Continuous variables are presented as mean ± standard error (SE) and compared using ANCOVA adjusted for age and sex. Binary outcomes (OP and KOA) are presented as percentages (number of cases) and compared using the chi-square test. *p*-values indicate between-group significance. The χ^2^ test was used to compare categorical variables (prevalence of musculoskeletal outcomes (OP and KOA)) between groups. Variables marked with ^#^ remained significant after Bonferroni correction for multiple testing. Abbreviations: VDS, vitamin D sufficient; VDD, vitamin D deficient; BMI, body mass index; WHR, waist–hip ratio; FM/WT, fat mass/weight ratio; ASMM/WT, appendicular skeletal muscle mass/weight ratio; TG, triglycerides; TC, total cholesterol; TC/HDL-C, total cholesterol-to-high-density-lipoprotein-cholesterol ratio; IL-9, interleukin-9; IL-34, interleukin-34; MCP-1, monocyte chemoattractant protein-1; hs-CRP, high-sensitivity C-reactive protein; SII, systemic immune-inflammation index; OP, osteoporosis; KOA, knee osteoarthritis; NS, non-significant.

**Table 2:** Multivariable logistic regression analysis of independent predictors of VD status *.

VD Status (771 VDS vs. 304 VDD)			
Independent Covariate	OR (95% CI)	β (SE)	*p*-Value
Sex (females vs. males)	4.11 (2.56–6.59)	1.41 (0.24)	4.39 × 10^−8^
Age (years)	1.38 (1.14–1.66)	0.32 (0.09)	0.0005
FM/WT	1.37 (1.09–1.72)	0.31 (0.11)	0.006

* Odds ratios (OR) with 95% confidence intervals (CI) and corresponding beta coefficients (β) with standard errors (SE) are shown. At the initial stage of the study, the following independent variables were tested stepwise forward: Age, sex, and FM/WT. All the variables in the analysis were standardized prior to statistical analysis. Abbreviations: VD, vitamin D; FM/WT, fat mass/weight ratio; VDD, vitamin D deficient; VDS, vitamin D sufficient.

**Table 3:** Multiple linear regression analysis of plasma IL-34 levels and VD status, age, and sex as predictors *.

Dependent Variable: Serum IL-34			
Independent Variables	Standardized β	SE of β	*p*-Value
Sex (females vs. males)	−0.006	0.049	NS
Age (years)	−0.090	0.048	NS
VD	0.529	0.049	6.99 × 10^−23^

* Standardized beta coefficients (β) with standard errors (SE) are shown. Abbreviation: IL-34, interleukin-34. VD, vitamin D; NS, non-significant.

**Table 4:** Associations between VD status, OP, and KOA as assessed by the chi-square test and Phi coefficient *.

Pair	χ^2^	Phi Coefficient	*p*-Value
VD × OP	38.11	0.19	<0.00001 **
VD × KOA	43.15	0.20	<0.00001 **
OP × KOA	21.07	0.14	<0.00001 **

* Associations between binary traits were examined using Pearson’s chi-square test. The Phi coefficient is presented as a measure of the strength of association. ** Associations remained statistically significant after adjustment for age and sex. Abbreviation: χ^2^ chi-square statistics; VD, vitamin D; OP, osteoporosis; KOA, knee osteoarthritis.

## Data Availability

The original contributions presented in this study are included in the article. Further inquiries can be directed to the corresponding author.

## Abbreviations

The following abbreviations are used in this manuscript (in alphabetical order):

ASMM/WT	Appendicular skeletal muscle mass/weight ratio
BIA	Bioelectrical impedance analysis
BMI	Body mass index
FM/WT	Fat mass/weight ratio
HDL-C	High-density lipoprotein cholesterol
Hs-CRP	High-sensitivity C-reactive protein
IL-9	Interleukin-9
IL-34	Interleukin-34
KOA	Knee osteoarthritis
MCP-1	Monocyte chemoattractant protein-1
OA	Osteoarthritis
OP	Osteoporosis
SII	Systemic immune-inflammation index
TC	Total cholesterol
TC/HDL-C	Total cholesterol to HDL-C ratio
TG	Triglycerides
VD	Vitamin D
VDD	Vitamin D deficiency
VDS	Vitamin D sufficiency
WHR	Waist–hip ratio
